# Machine learning to predict lymph node metastasis in T1 esophageal squamous cell carcinoma: a multicenter study

**DOI:** 10.1097/JS9.0000000000001694

**Published:** 2024-06-21

**Authors:** Xu Huang, Qingle Wang, Wenyi Xu, Fangyi Liu, Liangwei Pan, Heng Jiao, Jun Yin, Hongbo Xu, Han Tang, Lijie Tan

**Affiliations:** a Department of Thoracic Surgery, Zhongshan Hospital, Fudan University Shanghai, China; b Departments of Radiology, Zhongshan Hospital, Fudan University Shanghai, China; c The School of Basic Medical Sciences, Fudan University, Shanghai, China; d Department of Cardiothoracic Surgery, Lu’an Affiliated Hospital of Anhui Medical University, Lu’an, People’s Republic of China

**Keywords:** endoscopic mucosal resection, endoscopic submucosal dissection, esophageal squamous cell carcinoma, superficial cancer, T1

## Abstract

**Background:**

Existing models do poorly when it comes to quantifying the risk of lymph node metastases (LNM). This study aimed to develop a machine-learning model for LNM in patients with T1 esophageal squamous cell carcinoma (ESCC).

**Methods and results:**

The study is multicenter and population based. Elastic net regression (ELR), random forest (RF), extreme gradient boosting (XGB), and a combined (ensemble) model of these were generated. The contribution to the model of each factor was calculated. The models all exhibited potent discriminating power. The elastic net regression performed best with an externally validated the area under the curve (AUC) of 0.803, whereas the NCCN guidelines identified patients with LNM with an AUC of 0.576 and the logistic model with an AUC of 0.670. The most important features were lymphatic and vascular invasion and depth of tumor invasion.

**Conclusions:**

Models created utilizing machine learning approaches had excellent performance estimating the likelihood of LNM in T1 ESCC.

## Introduction

Highlights

- This study generated elastic net regression (ELR), random forest (RF), extreme gradient boosting (XGB), and a combined (ensemble) model to quantify the risk of lymph node metastases (LNM) in patients with T1 esophageal squamous cell carcinoma, which existing models do poorly.
- The models all exhibited potent discriminating power and the elastic net regression model performed best, lymphatic and vascular invasion and depth of tumor invasion are the most important.
- The model created utilizing machine learning approaches might be used to determine, which patients require additional surgery after endoscopic resection of T1 esophageal squamous cell carcinoma.

Esophageal cancer (EC) is one of the most aggressive gastrointestinal cancers and has a poor outcome. Worldwide, EC is the sixth most common cause of cancer death^1^. The majority of ECs in China are esophageal squamous cell carcinomas (ESCC), which account for more than 90% of all esophageal malignancies^2^. Endoscopic mucosal dissection maintains the integrity of the esophagus and lowers the procedure’s morbidity and mortality as compared to major surgical resection^3^. However, endoscopic therapy is only effective for EC with a minimal likelihood of lymph node metastases (LNM). While for patients with LNM, surgery with radical lymph node dissection is essential to reduce recurrence and extend survival^4–6^. Therefore, developing a tool that precisely predicts LNM in patients with T1 ESCC is critical.

Several risk prediction models for T1 EC LNM have been developed^7,8^. However, almost all prediction models are based on logistic regression models with a small sample size, poor precision, and generally lack of validation. As a branch of artificial intelligence, machine learning (ML) algorithms have developed and become more prominent for their increased versatility in capturing complicated nonlinear correlations, which are challenging to model effectively with logistic approaches^9–11^. Though ML may outperform traditional techniques in predictive performance, up to now, no individualized ML prediction model aimed at predicting LNM in T1 esophageal squamous cell carcinoma patients is available. In this study, we used ML algorithms to develop and validate clinically useful predictive models.

## Methods

### Study population

Patients who underwent esophagectomy at the Department of Thoracic Surgery from our high-capacity center for histologically confirmed pT1 ESCC between January 2010 and September 2019 were identified. The inclusion criteria were: (I) thoracic ESCC, (II) no history of concomitant or prior malignancy, (III) tumor with pT1 staging, (IV) 15 or more lymph nodes examined. After excluding patients who underwent neoadjuvant treatment or endoscopic submucosal dissection before surgery, 926 patients were included in the derivation cohort.

The internal-external validation cohorts comprised 205 patients who underwent esophagectomy for pT1 ESCC at the Department of Thoracic Surgery from our high-capacity center between September 2020 and September 2021. The external validation cohort consisted of 136 patients at the Department of Thoracic Surgery from two other high-capacity centers who underwent esophagectomy for pT1 ESCC between September 2015 and September 2021. The same inclusion and exclusion criteria were performed when identifying patients from the same prospectively collected database.

The institutional review board of our center, Fudan University, approved the use of a prospectively maintained database of patients with ESCC for this retrospective study.

### Clinicopathological data

The clinicopathological data of enrolled patients were collected from the database. All surgically resected specimens were evaluated by pathologists according to the 8th edition of the American Joint Committee on Cancer (AJCC) staging system. The following factors were collected: each patient’s age and sex, as well as depth of tumor invasion, tumor size, tumor location, macroscopic tumor type, cN stage, cT stage, lymph vascular invasion, histologic grade, numbers of lymph nodes dissected, and LNM. Each patient’s age, sex, tumor location, and macroscopic tumor type were extracted from electronic or paper health record systems and reviewed retrospectively. And the macroscopic tumor type was classified as flat type or nonflat type according to the previous study^12^. Clinical tumor staging was carried out based on preoperative endoscopic biopsies, endoscopic ultrasonography, and the chest and abdomen CT or PET-CT findings, according to the 8th edition of the American Joint Committee on Cancer (AJCC) staging system. Complete resection was classified as R0 resection, while incomplete resection with microscopic remaining disease was classified as R1, with macroscopic remaining R2. Resection specimens were histopathologically evaluated in accordance with WHO standards. Immunostaining, hematoxylin and eosin (HE)-stained specimens were used to examine the histologic grade and lymph vascular invasion.

### Surgery

The standard surgical approach included open or minimally invasive esophagectomy. Details have been described in a previous publication. Most patients in this study underwent two-field lymphadenectomy and extensive lymphadenectomy along the bilateral recurrent laryngeal nerves, which had been the authors’ regular procedure. Three-field lymphadenectomy was performed in a few patients because of a suspicion of cervical lymphadenopathy. All resection specimens were assessed by two experienced pathologists and staging was done using the eighth edition of the AJCC staging classification^14^. The resected specimens were fixed on cork and immersed in 10% formalin. The whole specimens were cut into 2 mm thick sections and examined. Tumor size, vessel invasion, grade of differentiation and resection margins were examined histopathologically. Tumor invasion to the mucosal layer was defined as T1a, and invasion to the submucosa as T1b.

### Predictor characteristics

The characteristics included clinical variables (age and sex, depth of tumor invasion, tumor size, tumor location, macroscopic tumor type, lymph vascular invasion, and histologic grade) and preoperative hematologic indices ( AFP, CEA, CA19.9, squamous epithelial cell antigen, and cytokeratin 19). Based on the logistic variable screening results and evidence provided in some previous literature and its practicality and relevance in clinical practice, machine-learning models were developed using data on patients’ age and sex, depth of tumor invasion, tumor size, tumor location, macroscopic tumor type, lymph vascular invasion, and histologic grade (Supplementary eTable 2, Supplemental Digital Content 1, http://links.lww.com/JS9/C811)^7,13^. Most of our data were structured as binary features except for age and tumor size. Location was three categorical variables. Age and tumor size were normalized to be in a range [0–1]. The Fisher exact test and the Kolmogorov–Smirnov test were used for assessing categorical variables or continuous variables between groups. Statistical significance was set at *P*<0.05.

### ML model building and validation

Elastic net regularized logistic regression (ELR)^14^ and random forest (RF)^15^ and extreme gradient boosting (XGB)^16^ were applied to predict LNM. ELR mixes the penalties of ridge and lasso, which is beneficial for dealing with high-dimensional data and feature selection^14,17^. In the context of cancer research, ELR has been shown to improve the predictive accuracy of models by reducing overfitting and selecting the most relevant features^9^. RF combines a predetermined number of decision trees (usually about 1000) generated on a random subgroup of the data set. In cancer prognostication, it has been widely applied due to its high accuracy and ability to quantify feature importance^18^. XGB aims to improve consecutively. XGB aims to improve consecutively by creating models to explain where the previous model fails and then repeating this process (usually around 1000 times). At the same time, regularization is used to reduce overfitting. In the field of oncology, XGB has been recognized for its capacity to capture complex, nonlinear relationships and improve predictive accuracy^19^. Therefore, we chose the method to leverage its strengths in handling complex datasets and its ability to refine predictions based on the residual errors of previous models. The individual models were combined to generate overall predictions^20^. This strategy, theoretically, is advantageous when utilizing a variety of model types that represent various aspects of patients’ risk profiles.

Random over sampling examples (ROSE) was applied to deal with the class imbalance problem^21^. During model creation, a 10-fold cross-validation with five repeats was used for each model’s hyperparameter tuning. And log loss was used as an optimization metric. For ELR, the hyperparameter tuning was conducted for α and λ hyperparameters. Thousand decision trees were used to create the RF model. The split rule, minimum node size, and number of variables per tree were all hyperparameter tuned. The hyperparameters for the XGB model include maximum tree depth, number of optimization rounds, minimum weight in each child node, minimum loss reduction (γ), regularization penalty (η), and subsampling for regularization. These three best-performed models were then integrated to create the ensemble model (Ens) by applying logistic regression to create a linear blend of projected probabilities.

The area under the receiver operator characteristic (ROC) curve (AUC) was used to evaluate the models’ discrimination power. Calculated the accuracy, sensitivity, precision, and F1 score of several models as auxiliary indicators for judging predictive performance. Internal validation was carried out with 1000 resampled datasets and a 0.632 bootstrapping strategy. Calibration was assessed visually and formally with the Hosmer–Lemeshow test. Isotonic regression was used to scale probabilities on the final model to enable a meaningful interpretation of probability.

The VarImp function of the caret R package was used. The contribution of each variable to the global ROC curve was calculated as a percentage.

### Sub-analysis

The diagnostic ability of the ML model was compared with the NCCN guidelines for LNM. In the NCCN guidelines, poorly differentiated tumors, deep submucosal invasion, and lymphovascular invasion are considered predictive of LNM.

Data analysis was conducted using R version 3.5.3 (R Foundation for Statistical Computing). The caret and caretEnsemble packages were used to train the models. The full R code to train the models is available in Supplementary, along with a list of packages used (Supplemental Digital Content 1, http://links.lww.com/JS9/C811).

The calibrated final model was designed using R Shiny (available freely at https://predicted.shinyapps.io/Rshinyfinal/). No data entered into the model were collected or stored.

The work has been reported in line with the strengthening the reporting of cohort, cross-sectional, and case–control studies in surgery (STROCSS) criteria (Supplemental Digital Content 1, http://links.lww.com/JS9/C812)^22^.

## Results

### Patient characteristics

A total of 926 patients were included in the training set, 205 patients and 136 patients were enrolled in the internal-external validation cohort and external validation cohort. Table 1 shows the comparative data of patients between the training and validation cohorts. LNM were present in 139 of 926 patients (15.01 percent) and 48 of 341 patients (14.10 percent) with no statistical difference between the derivation and validation cohorts (*P*>0.05). In the training set, patients with LNM were significantly associated with tumor location, macroscopic tumor type, length of tumor, pT stage, and lymph vascular invasion(*P*<0.05) (Supplementary eTable 1, Supplemental Digital Content 1, http://links.lww.com/JS9/C811).

**Table 1:** Clinical and pathological characteristics of patients in the training and validation cohorts and comparison of significance difference.

	Overall	Derivation	Validation	
	*N*=1267	*N*=926	*N*=341	*P*
Age				
<65	769 (60.7)	557 (60.2)	212 (62.2)	0.557
≥65	498 (39.3)	369 (39.8)	129 (37.8)	
Sex				
Female	332 (26.2)	249 (26.9)	83 (24.3)	0.399
Male	935 (73.8)	677 (73.1)	258 (75.7)	
Tumor location				
Lower	476 (37.6)	374 (40.4)	102 (29.9)	0.001
Middle	686 (54.1)	471 (50.9)	215 (63.0)	
Upper	105 (8.3)	81 (8.7)	24 (7.0)	
Macroscopic tumor type				
Nonflat	413 (32.6)	257 (27.8)	156 (45.7)	<0.001
Flat	854 (67.4)	669 (72.2)	185 (54.3)	
Histological grade				
Poor	219 (17.3)	158 (17.1)	61 (17.9)	0.806
Moderate	773 (61.0)	570 (61.6)	203 (59.5)	
Well	275 (21.7)	198 (21.4)	77 (22.6)	
Length of tumor [IQR]	1.7 [1.2–2.3]	1.7 [1.2–2.5]	1.5 [1.0–2.1]	0.059
pT (%)				
pT1a	388 (30.6)	270 (29.2)	118 (34.6)	0.072
pT1b	879 (69.4)	656 (70.8)	223 (65.4)	
pN (%)				
−	1080 (85.2)	787 (85.0)	293 (85.9)	0.744
pN (%)				
+	187 (14.8)	139 (15.0)	48 (14.1)	
LVI (%)*				
−	1086 (85.7)	787 (85.0)	299 (87.7)	0.261
+	181 (14.3)	139 (15.0)	42 (12.3)	

* Values are presented as the patient numbers (*n*, %) or medians [IQR].

LVI, lymph vascular invasion; pN, pathological lymph node stage; pT, pathological tumor stage.

### Model performance: discrimination

**Figure 1:**
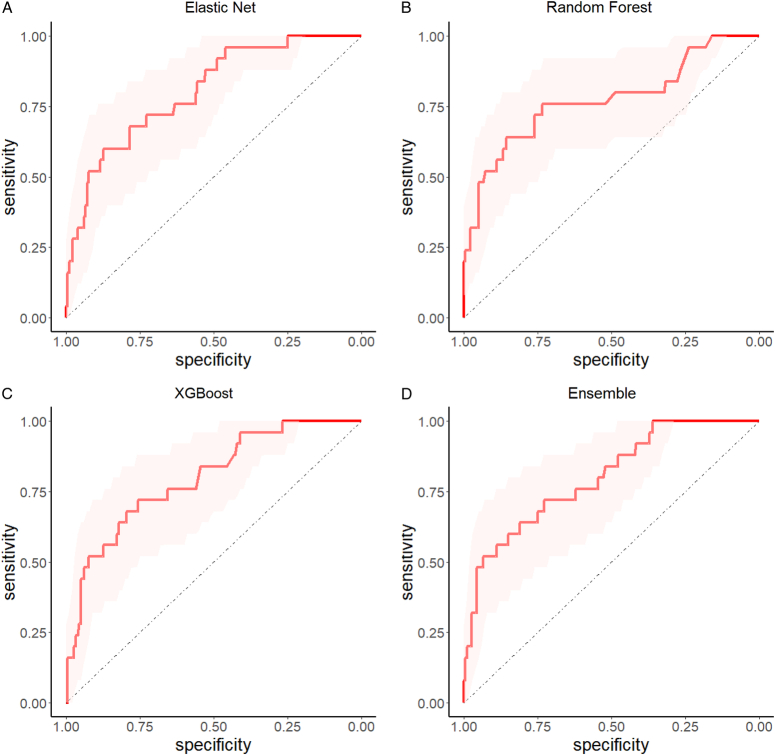
ROC curve of Elastic Net (A), Random Forest (B), XGBoost (C), and Ensemble (D) in the internal-external valid cohort.

All four models demonstrated excellent discrimination both in the training cohort and valid cohorts. The data are shown in Table 2, Figure 1 and Supplementary eFigs. 1–3 (Supplemental Digital Content 1, http://links.lww.com/JS9/C811). In the train cohort, the RF showed the best discrimination capacity with an AUC of 0.838, followed by Ens (0.822) and ELR (0.814). The XGB had the worst performance with an AUC of 0.803. In the internal and internal-external cohorts, the Elastic Net showed the best performance (AUC 0.800 in the internal cohort and 0.803 in the internal-external cohort) as compared with XGB (AUC 0.798 in the internal cohort and 0.791 in the internal-external cohort) and RF (AUC 0.794 in the internal cohort and 0.773 in the internal-external cohort). The ensemble model did not show a better AUC (0.797 in the internal cohort and 0.799 in the internal-external cohort) compared with EL. In general, only the AUC of EL in the three main groups are all more than 80%, which means the best model discrimination performance. In the external validation cohort, the AUC of the four models is also close to 0.8 (0.787 for EL, 0.766 for RF, 0.783 for XGB, 0.790 for Ens), meaning that the model has good universality.

**Table 2:** The discrimination of four models based on AUC.

		Area in the curve		
	Train	Internal validation	Internal-external validation	External validation
Elastic net regression	81.4% (77.4–85.4%)	80.0% (75.8–84.1%)	80.3% (71.3–89.3%)	78.7% (67.9–89.5%)
Random forest	83.8% (80.3–87.3%)	79.4% (75.3–83.5%)	77.3% (65.7–88.9%)	76.6% (62.7–90.5%)
XGBoost	80.3% (76.1–84.5%)	79.8% (75.6–84.0%)	79.1% (70.1–88.1%)	78.3% (67.2–89.5%)
Ensemble	82.2% (78.3–86.2%)	79.7% (77.8–81.6%)	79.9% (70.7–89.1%)	79.0% (68.4–89.7%)

Values in parentheses are 95% CIs.

Supplementary eTable 3 (Supplemental Digital Content 1, http://links.lww.com/JS9/C811) provides the accuracy, sensitivity, precision and F1 score of several algorithms in the training set, internal and external validation sets, and external validation sets. ML algorithms have shown excellent predictive performance, with EL being the most balanced indicator, consistent with our ROC curve.

### Model performance: calibration

Throughout visually observing, calibration was best in the ELR, and the worst in the XGB. The Hosmer–Lemeshow test confirmed the result (*P*=0.3102 for El, *P*=0.1051 for Ens, *P*=0.5697 for RF, *P*<0.01 for XGB). In terms of EL, the calibration before and after scaling can be observed in Supplementary eFig 4 (Supplemental Digital Content 1, http://links.lww.com/JS9/C811). The Hosmer–Lemeshow test gave a *χ* ^2^ value of 325.336 (*P*<0⋅001) before and 9.3931 (*P*=0⋅3102) after scaling.

### Variable importance

**Figure 2:**
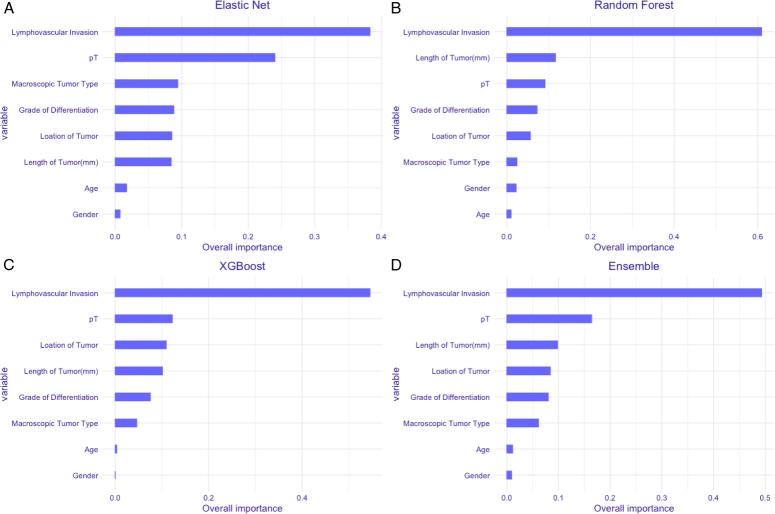
The variable importance of Elastic Net (A), Random Forest (B), XGBoost (C) and Ensemble (D).

Feature importance analysis was assessed to learn about the contribution of each variable to the models. Overall, in four models, the LVI was the most influential predictor, followed by depth of tumor invasion (pT stage). In terms of other variables, there were considerable differences in models. (Supplementary eTable 4, Supplemental Digital Content 1, http://links.lww.com/JS9/C811, Fig. 2). For example, tumor differentiation contributed 8.8% to the ELR model, 7.2% to the RF model, 7.5% to the XGB model, and 8.0% to the ensemble model.

### Sub-analysis (ML Model vs. Logistic Model vs. NCCN Guideline)

**Figure 3:**
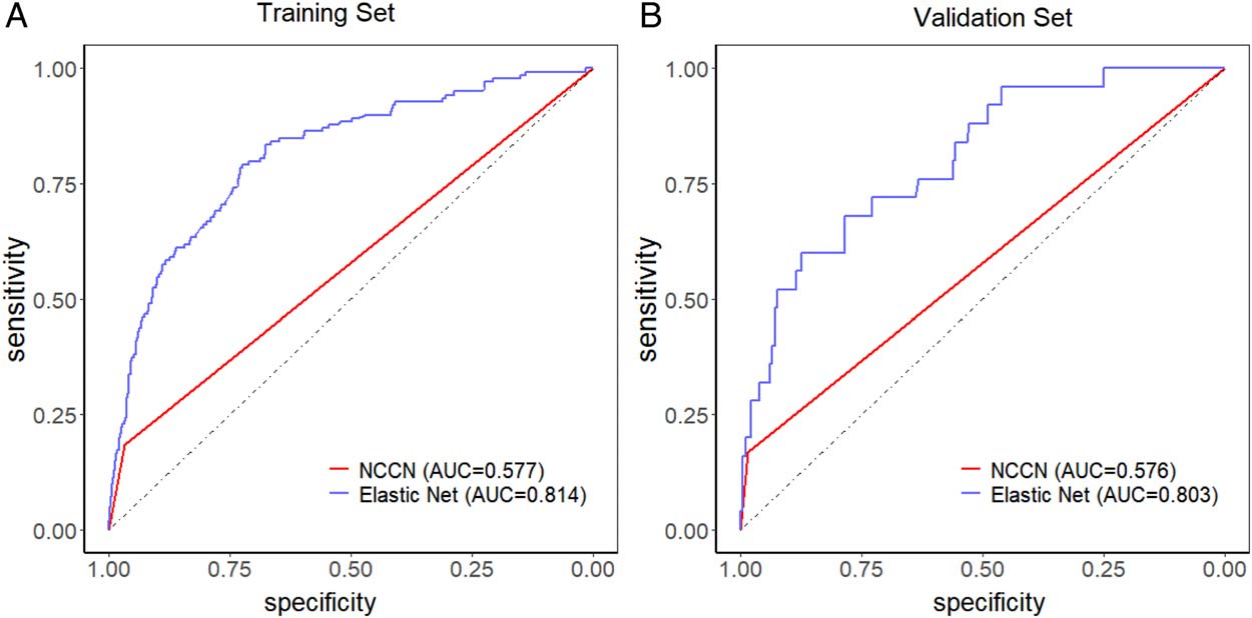
The comparison between the Elastic Net and NCCN in the train cohort (A) and validation cohort (B).

Results showed that the EL model (AUC: 0.814 in the training cohort; 0.803 in the internal-external validation cohort) showed higher performance than the NCCN guidelines. (AUC: 0.576 in the training cohort; 0.575 in the internal-external validation cohort) (Fig. 3).

Compared to the logistic (AUC:0.865 in the training cohort; 0.670 in the internal-external validation cohort), our ML model exhibited better predictive performance (Supplementary eFig. 5, Supplemental Digital Content 1, http://links.lww.com/JS9/C811).

## Discussion

In this study, we developed and validated four ML models to predict LNM in patients with pT1 ESCC. To the best of our knowledge, this is the largest study to explore the use of ML in assessing the risk of LNM of pT1 ESCC. Our results demonstrate that ML can accurately predict LNM in T1 ESCC and may be beneficial in determining whether additional surgical resection is necessary after endoscopic resection of pT1 ESCC.

This study is a retrospective study, primarily due to the availability of a large-scale, well-defined cohort of T1 ESCC patients treated by three institutions within a decade. This design enables us to effectively utilize existing data to develop and validate predictive models for LNM in this specific patient population. In addition, we also considered the predictive utility of the predictive variables and their applicability in practical applications. We focus on clinically evaluated pathological variables in clinical practice, which have previously been reported to be associated with LNM in ESCC, such as tumor infiltration depth, lymphatic vessel infiltration, and tumor differentiation. The addition of these ready-made factors enhances the practical applicability of our model, as they can be easily integrated into clinical decision-making processes without the need for additional testing or procedures.

LN status is a crucial prognostic factor, and the metastasis of LN (LNM) usually indicates a poor prognosis, which emphasizes the necessity of radical lymph node dissection after esophagectomy. However, for patients with early ESCC, especially pT1 ESCC, LNM rarely occurs. In contrast to extensive surgical resection, endoscopic mucosal dissection preserves the integrity of the esophagus^3^. While the curativeness of endoscopic resection depends on the risk of lymph node metastasis. At present, preoperative assessment of LNM in patients with ESCC generally depends on CT images through LN size criteria. However, the discrimination power of CT has been questioned in previous studies^23^. The lymph node biopsy, EUS, and PET-CT have exhibited varying differentiation ability in LNM recognition, and invasive or expensive procedures remain to be doubted^24^. Therefore, a precise and noninvasive tool distinguishing LNM preoperatively is warranted.

The current methods for constructing LNM prediction models in early ESCC are mainly based on logistic regression, and multivariate analysis of clinicopathological characteristics is carried out to confirm the risk factors for variable selection. Several studies developed nomograms with fairly favorable risk stratification in early-stage ESCC LNM^7,8^. In traditional logistic models built in the past, the prediction models’ AUC was usually between 0.65 and 0.70, and most logistic models are accompanied by overfitting^19^. Similarly, our previous study showed high performance (The C-index was 0·790 in the derivation cohort and 0·789 for the validation cohort)^7^. In our research, though logistics had high performance in the training cohort, it only exhibits 0.670 AUC in our internal-external validation cohort, which indicates a serious overfitting. The literature on ML prediction models based on clinical factors for LNM of T1 ESCC is limited. In previous studies, Li *et al*. endeavored to employ the naive Bayes algorithm to predict the LNM of T1-T2 stage ESCC. However, their study was constrained by a limited sample size, with only 519 T1 stage cases in total. The AUC in the training set and external validation set were 0.715 and 0.752, respectively^25^. Chen *et al*.^26^ built an artificial neural network with 733 patients based on clinical features for superficial esophageal squamous cell carcinoma (SESCC). However, a concern demonstrated in their work is the lack of external testing regarding the predictive performance of their models. Our study is the largest to date that constructs robust LNM prediction models for T1 stage ESCC patients using ML algorithms. We utilized a large sample size and performed comprehensive comparisons of various ML models with convincing validation of our results. We also compared ML algorithms with traditional logistic regression models and NCCN guidelines, further demonstrating the advantage of our models. Our results demonstrate the superiority of ML approaches, particularly the ELR model, which achieved an AUC of 0.803 in the validation cohort, surpassing the performance of logistic regression (AUC: 0.670) and NCCN guidelines (AUC: 0.575). The superior predictive power and practical applicability of our ML models highlight their potential to significantly improve clinical decision-making and patient outcomes in the management of early-stage EC.

Another strength of our study is that we pioneered the evaluation of the contribution of different variables to lymph node metastasis in esophageal squamous cell carcinoma using various ML models. The most important features in the LNM prediction models were lymphovascular invasion (LVI), which accounted for 42.6% of performance in the final model. Studies have confined that the probability of metastasis is generally accompanied by an advanced degree of LVI^27,28^. Our findings are in agreement with those of previous studies showing that LVI was strongly associated with LNM. Submucosa is considered as the boundary layer of EC cell metastasis^29^. Previous studies have reported that the rate of LNM increases with increasing depth of tumor infiltration^30,31^. However, relying solely on LVI to determine whether LNM has occurred may not be accurate. Linear NCCN guidelines typically classify cases of deep submucosal infiltration, poorly differentiated tumors, or LVI as LNM. In our study, we found that the AUC and F1 scores of the guidelines were significantly lower than those of our ML model, which may indicate that relying on a single indicator to judge results is not ideal, and other factors that may be related to LNM should not be ignored. While some variables may have lower importance scores compared to LVI, their inclusion in the models enhances the overall predictive performance and provides additional granularity in risk stratification. In addition, as shown in the variable contribution graph presented in our web calculator, quantifying the promoting or mitigating effects of each variable on LNM may be more conducive to guiding personalized treatment decisions and risk assessment for T1 esophageal squamous cell carcinoma patients, enhancing the clinical effectiveness and applicability of the study.

The main strengths of this study were an adequate training database and credible validation of different prediction models, leading to convincing results and potentially promising value in clinical practice. The limitations of the study are also obvious. First, potential selection bias was unavoidable as it was a retrospective-designed study. Second, the clinicopathological factors included in ML models were insufficient to some extent. For example, mucosa and submucosa could not be classified into subgroups such as SM1, SM2, and SM3 during the analyses because of insufficient data. Prospective studies on larger multi-institutional cohorts are needed in the future, and additional predictive factors need to be included to validate and improve our model.

## Conclusions

Four ML models were generated based on clinicopathological features for predicting LNM in T1 ESCC. All models exhibited superior working performance, while EL models performed the best. The EL models showed higher discriminating power than the current NCCN guidelines to predict LNM in patients with pT1 ESCC. Lymphatic and vascular invasion (LVI) and depth of tumor invasion (pT) were the most significant variables. Application of ML algorithms in LNM prediction of early ESCC patients is clinically practical.

## Ethical approval

The study was conducted according to the Declaration of Helsinki and approved by the ethics committee of Zhongshan Hospital (B2021-322). Informed consent was obtained from all the patients.

## Consent

Not applicable.

## Source of funding

This work was supported by the National Natural Science Foundation of China (Grant Number: 81902396), and Zhongshan Hospital (Grant Number: 2020ZSLC5 and 2021ZSYQ27).

## Author contribution

L.T. and H.T.: concept and design; X.H., Q.W., H.X., F.L., L.P., W.X., H.T., L.T., H.J., and J.Y.: acquisition, analysis, or interpretation of data; X.H., Q.W., and X.: drafting of the manuscript; X.H., Q.W., X., F.L., L.P., X., H.T., L.T., H.J., and X.: critical revision of the manuscript for important intellectual content; X.H., Q.W., X., and F.L.: statistical analysis and model design; H.T. and L.T.: obtained funding; X.H., Q.W., X., F.L., L.P., X., H.T., L.T., H.J., and J.Y.: administrative, technical, or material support; X.H., Q.W., X., F.L., L.P., X., H.T., L.T., H.J., and J.Y.: supervision.

## Conflicts of interest disclosure

The authors declares no conflicts of interest.

## Research registration unique identifying number (UIN)

Trial registration number of the research is NCT06256185.

## Guarantor

Lijie Tan.

## Data availability statement

Any datasets generated during and analyzed during the current study are available upon reasonable request.

## Provenance and peer review

Not applicable.

## Presentation

Not applicable.

## Supplementary Material

SUPPLEMENTARY MATERIAL
