# Supplementary material for: Machine learning to predict lymph node metastasis in T1 esophageal squamous cell carcinoma: a multicenter study
Source: Int J Surg. 2024 Jun 21;110(12):7852–9. doi: 10.1097/JS9.0000000000001694 (PMC11634146; doi:10.1097/JS9.0000000000001694)
Supplement: SUPPLEMENTARY MATERIAL [file js9-110-7852-s001.docx]

**Supplemental Tables**

**eTable 1 Comparison of significant differences in various clinical indicators of patients classified according to pN in the training and validation cohorts**

|  |  |  | training set |  |  |  | validation set |  |  |
| --- | --- | --- | --- | --- | --- | --- | --- | --- | --- |
|  |  | **Overal**  **N=926** | **-**  **N=787** | **+**  **N=139** | **P-Value** | **Overall**  **N=341** | **-**  **N=293** | **+**  **N=48** | **P-Value** |
| **age** | <65 | 557 (60.2) | 478 (60.7) | 79 (56.8) | 0.44 | 212 (62.2) | 184 (62.8) | 28 (58.3) | 0.667 |
|  | >=65 | 369 (39.8) | 309 (39.3) | 60 (43.2) |  | 129 (37.8) | 109 (37.2) | 20 (41.7) |  |
| **sex** | female | 249 (26.9) | 208 (26.4) | 41 (29.5) | 0.517 | 83 (24.3) | 75 (25.6) | 8 (16.7) | 0.248 |
|  | male | 677 (73.1) | 579 (73.6) | 98 (70.5) |  | 258 (75.7) | 218 (74.4) | 40 (83.3) |  |
| **tumor location** | lower | 374 (40.4) | 313 (39.8) | 61 (43.9) | 0.115 | 102 (29.9) | 76 (25.9) | 26 (54.2) | <0.001 |
|  | middle | 471 (50.9) | 410 (52.1) | 61 (43.9) |  | 215 (63.0) | 193 (65.9) | 22 (45.8) |  |
|  | upper | 81 (8.7) | 64 (8.1) | 17 (12.2) |  | 24 (7.0) | 24 (8.2) | 0 |  |
|  |  |  | training set |  |  |  | validation set |  |  |
|  |  | **Overal**  **N=926** | **-**  **N=787** | **+**  **N=139** | **P-Value** | **Overall**  **N=341** | **-**  **N=293** | **+**  **N=48** | **P-Value** |
| **Macroscopic Tumor Type** | non-flat | 257 (27.8) | 235 (29.9) | 22 (15.8) | 0.001 | 156 (45.7) | 141 (48.1) | 15 (31.2) | 0.044 |
|  | flat | 669 (72.2) | 552 (70.1) | 117 (84.2) |  | 185 (54.3) | 152 (51.9) | 33 (68.8) |  |
| **differentiation** | poor | 158 (17.1) | 140 (17.8) | 18 (12.9) | 0.305 | 61 (17.9) | 54 (18.4) | 7 (14.6) | 0.781 |
|  | moderate | 570 (61.6) | 483 (61.4) | 87 (62.6) |  | 203 (59.5) | 174 (59.4) | 29 (60.4) |  |
|  | well | 198 (21.4) | 164 (20.8) | 34 (24.5) |  | 77 (22.6) | 65 (22.2) | 12 (25.0) |  |
| **length of tumor [IQR]** | | 1.7 [1.2,2.5] | 1.5 [1.1,2.2] | 2.0 [1.5,3.0] | <0.001 | 1.5 [1.0,2.1] | 1.5 [1.0,2.0] | 1.9 [1.4,2.5] | 0.002 |
| **pT** | pT1a | 270 (29.2) | 249 (31.6) | 21 (15.1) | <0.001 | 118 (34.6) | 111 (37.9) | 7 (14.6) | 0.003 |
|  | pT1b | 656 (70.8) | 538 (68.4) | 118 (84.9) |  | 223 (65.4) | 182 (62.1) | 41 (85.4) |  |
|  |  |  | training set |  |  |  | validation set |  |  |
|  |  | **Overal**  **N=926** | **-**  **N=787** | **+**  **N=139** | **P-Value** | **Overall**  **N=341** | **-**  **N=293** | **+**  **N=48** |  |
| **LVI*** | no | 787 (85.0) | 720 (91.5) | 67 (48.2) | <0.001 | 299 (87.7) | 275 (93.9) | 24 (50.0) | <0.001 |
|  | yes | 139 (15.0) | 67 (8.5) | 72 (51.8) |  | 42 (12.3) | 18 (6.1) | 24 (50.0) |  |

* Values are presented as the patient numbers (n, %) or medians [IQR]. pT, pathological tumor stage; pN, pathological lymph node stage; LVI, lymph vascular invasion.

**eTable2 Selection of variables using logistic models**

| **Variable** |  | **OR (95%CI)** | **Pvalue** |
| --- | --- | --- | --- |
| **sex** | female | -0.02(-0.63~0.57) | 0.957 |
| **age** | >60 | 0.19(-0.35~0.74) | 0.492 |
| **tumor location** | middle | -0.2(-0.8~0.42) | 0.508 |
|  | upper | -0.36(-1.52~0.68) | 0.52 |
| **Macroscopic Tumor Type** | no flat | 0.36(-0.24~0.97) | 0.241 |
| **length of tumor** |  | 0.47(0.16~0.79) | 0.003 |
| **differentiation** | moderate | 0.2(-0.42~0.87) | 0.533 |
|  | well | 0.22(-0.67~1.1) | 0.622 |
| **pT** | pT1b | 1.06(0.29~1.95) | 0.011 |
| **LVI** | yes | 0.98(0.08~1.83) | 0.026 |
| **AFP** |  | 0.75(-2.9~4.18) | 0.676 |
| **CEA** |  | -0.37(-1.45~0.6) | 0.48 |
| **CA19.9** |  | -0.29(-1.44~0.58) | 0.569 |
| **Squamous.epithelial.cell.antigen** |  | 0.39(-0.1~1.02) | 0.169 |
| **Cytokeratin.19** |  | 0.38(-0.07~0.82) | 0.07 |

*LVI, lymph vascular invasion.

**eTable3 Accuracy, Sensitivity Precision and F1 Score of four models, NCCN and logistic**

|  | **sensitivity** | | | **precision** | | | **F1 score** | | | **accuracy** | | |
| --- | --- | --- | --- | --- | --- | --- | --- | --- | --- | --- | --- | --- |
|  | **train** | **internal-external validation** | **external validation** | **train** | **internal-external validation** | **external validation** | **train** | **internal-external validation** | **external validation** | **train** | **internal-external validation** | **external validation** |
| **ELastic Net** | 0.791 | 0.667 | 0.720 | 0.581 | 0.600 | 0.533 | 0.670 | 0.632 | 0.613 | 0.883 | 0.897 | 0.925 |
| **Random Forest** | 0.626 | 0.611 | 0.560 | 0.650 | 0.577 | 0.500 | 0.638 | 0.594 | 0.528 | 0.893 | 0.889 | 0.912 |
| **XGBoost** | 0.683 | 0.550 | 0.640 | 0.557 | 0.500 | 0.526 | 0.614 | 0.524 | 0.577 | 0.871 | 0.868 | 0.919 |
| **Ensemble** | 0.834 | 0.667 | 0.680 | 0.527 | 0.533 | 0.529 | 0.646 | 0.593 | 0.595 | 0.863 | 0.879 | 0.922 |
| **NCCN** | 0.949 | 0.944 | 0.960 | 0.186 | 0.187 | 0.168 | 0.311 | 0.312 | 0.286 | 0.367 | 0.449 | 0.415 |
| **logistic** | 0.486 | 0.320 | 0.333 | 0.667 | 0.444 | 0.600 | 0.562 | 0.372 | 0.428 | 0.888 | 0.868 | 0.882 |

**eTable4 the importance of each variable in the four models**

|  | **EL** | **RF** | **XGB** | **Ens** |
| --- | --- | --- | --- | --- |
| **age** | 0.017 | 0.010 | 0.003 | 0.011 |
| **sex** | 0.007 | 0.022 | 0.000 | 0.009 |
| **location** | 0.085 | 0.056 | 0.109 | 0.084 |
| **Macroscopic Tumor Type** | 0.094 | 0.024 | 0.046 | 0.061 |
| **differentiation** | 0.088 | 0.072 | 0.075 | 0.080 |
| **length of tumor** | 0.084 | 0.116 | 0.101 | 0.098 |
| **pT** | 0.240 | 0.091 | 0.122 | 0.164 |
| **LVI** | 0.383 | 0.609 | 0.545 | 0.493 |

*EL, Elastic net regression; RF, Random forest; XGB, XGBoost; Ens, Ensemble; pT, pathological tumor stage; LVI, lymph vascular invasion.

**Supplemental Figures**

**eFigure 1.ROC curve of Elastic Net(A), Random Forest(B), XGBoost(C) and Ensemble(D) in the train cohort.**


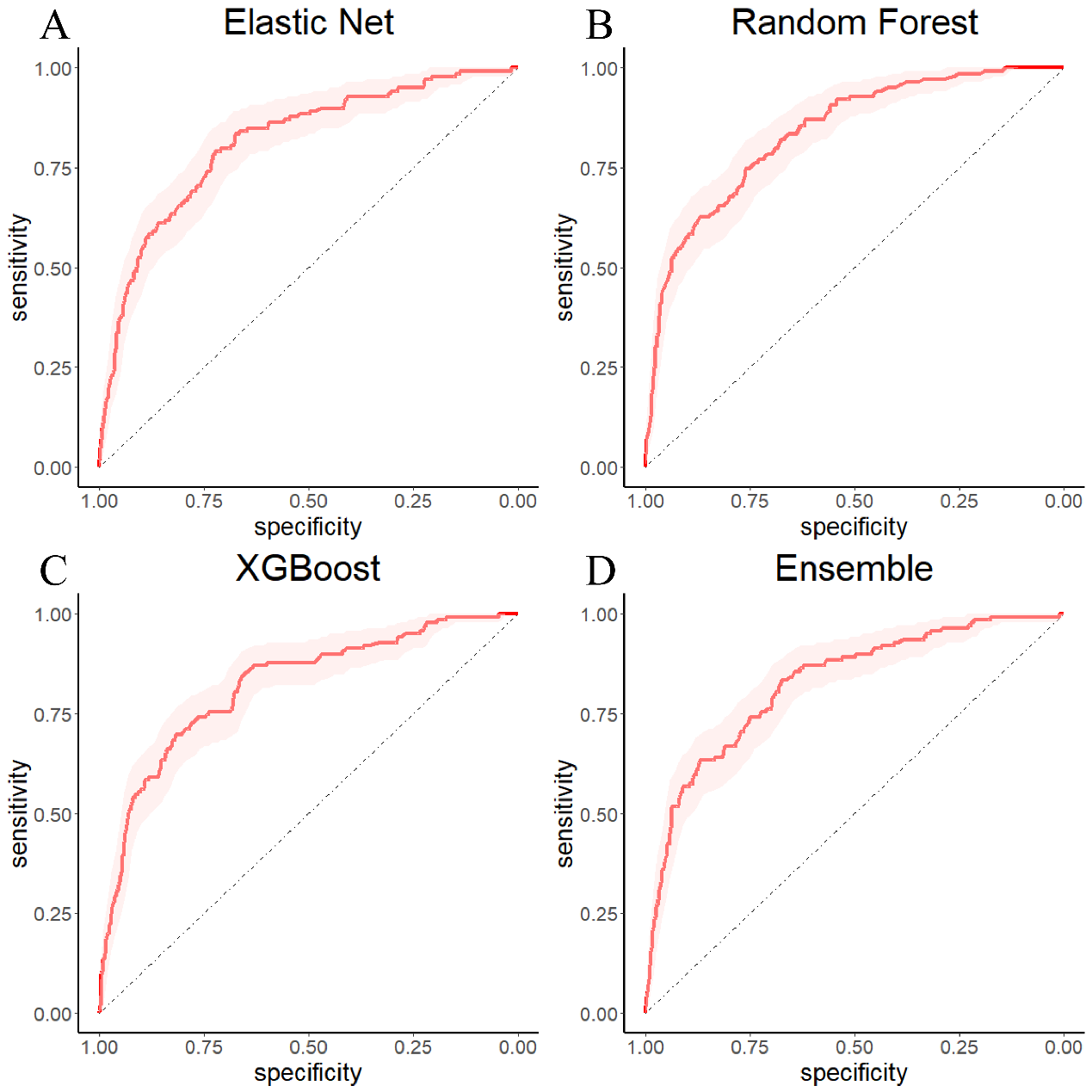


**eFigure 2.ROC curve of Elastic Net(A), Random Forest(B), XGBoost(C) and Ensemble(D) in the internal valid cohort.**


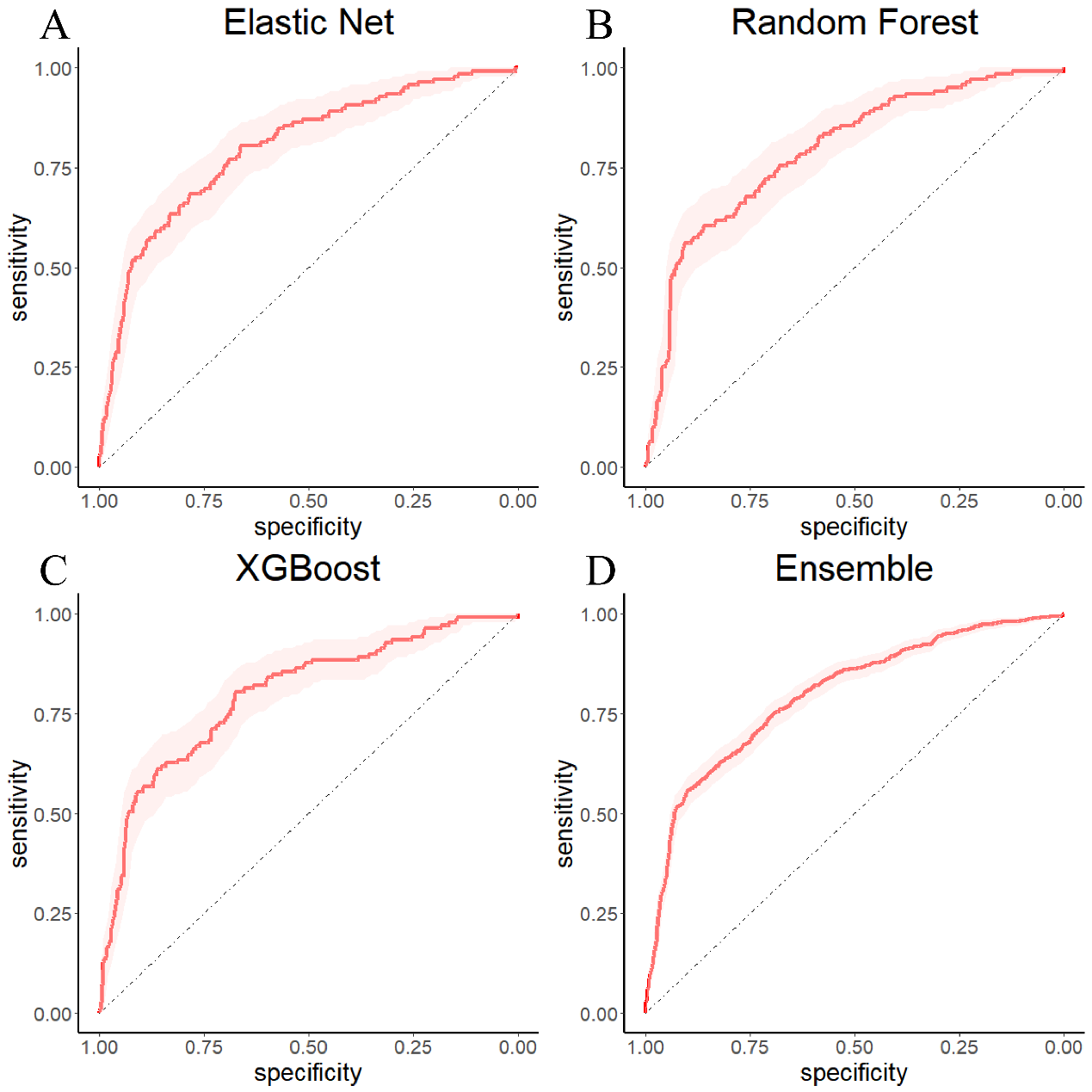


**eFigure 3.ROC curve of Elastic Net(A), Random Forest(B), XGBoost(C) and Ensemble(D) in the external valid cohort.**

**
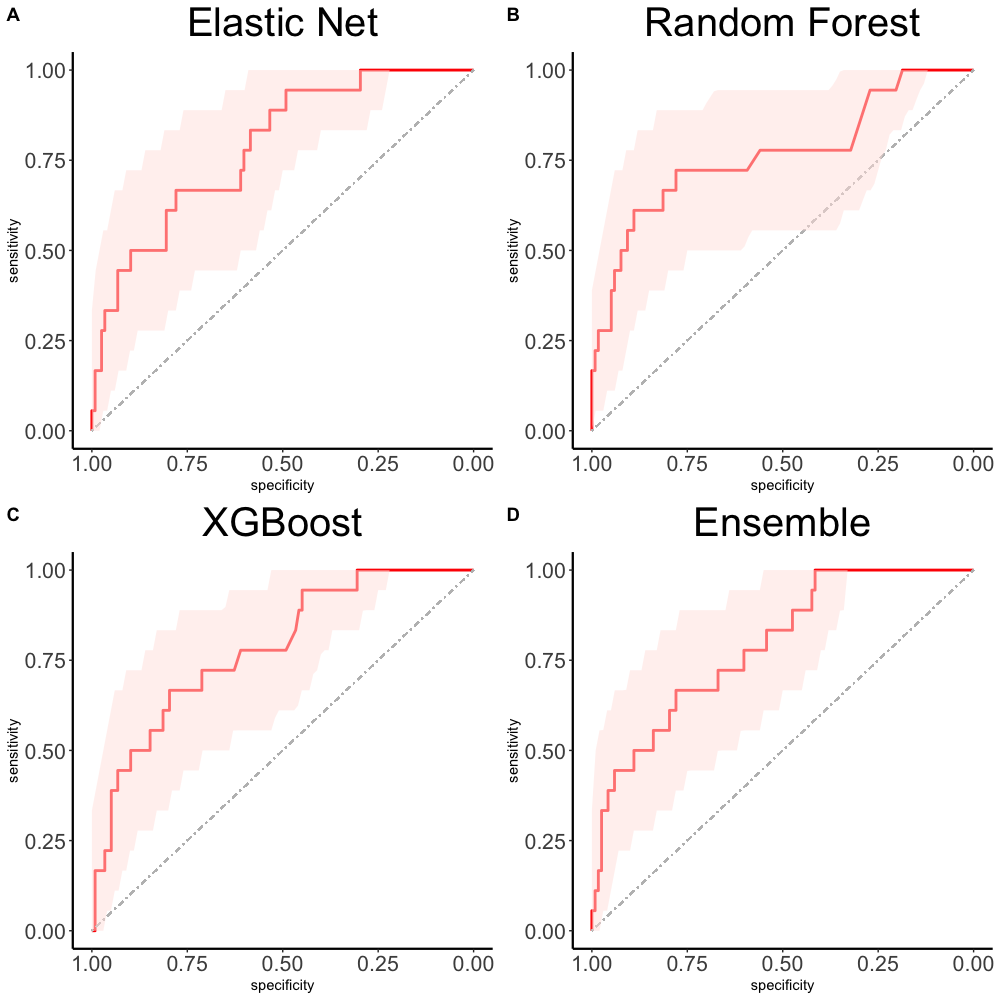
**

**eFigure 4 the calibration plot of the Elastic Net before calibrating(A) and after calibrating(B)**


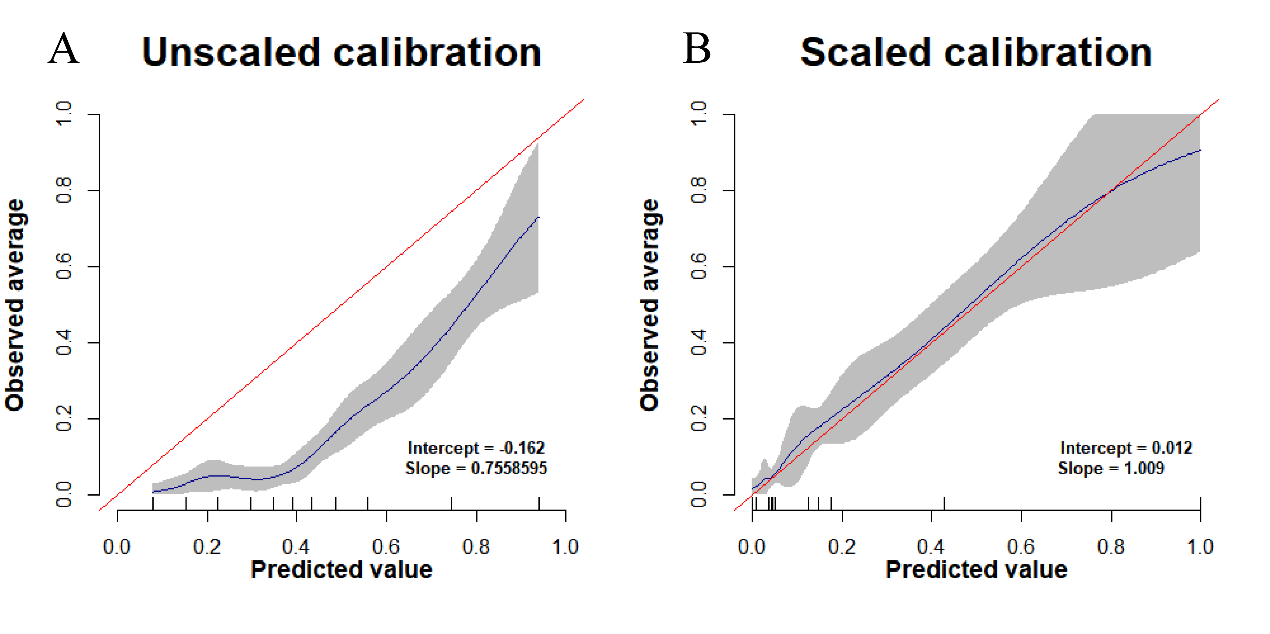


**eFigure 5.ROC curve of Elastic Net and logistic model in the training cohort(A) and in the validation cohort(B)**


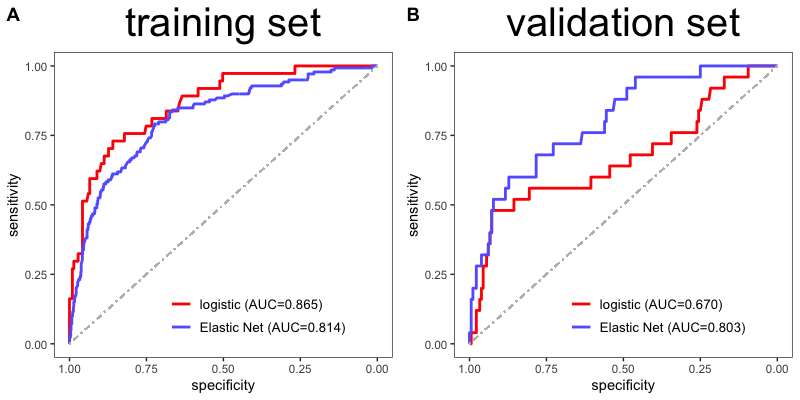


**Supplemental codes**

#install.packages("rsconnect")

library(tidyverse)

#install.packages("caret")

#install.packages("digest")

#install.packages("haven")

library(caret)

library(showtext)

library(plyr)

library(dplyr)

library(ggplot2)

library(ggpubr)

# core survival analysisfunctions

library(survival)

# recommended forvisualizing survival curves

library(survminer)

library(pROC)

library(ROCR)

library(car) #package to calculate Variance Inflation Factor

library(corrplot) #correlation plots

library(leaps) #best subsets regression

library(glmnet) #allows ridge regression, LASSO and elastic net

library(gbm) ###Generates Calibration plot

library(ResourceSelection)###Hosmer-Lemeshow test

#install.packages("data.table")

#install.packages("rlang")

library(rlang)

library(xgboost)

library(parallel)

library(foreach)

library(doParallel)

#install.packages("caretEnsemble")

library(caretEnsemble)###Combines models

library(tableone)

library(randomForest)

library(data.table)

#install.packages("SvyNom")

#install.packages("Matrix")

library(Matrix)

library(SvyNom)

# Step 1: Create the training dataset

dataCtr <- read.table("train.csv", header = TRUE,,encoding = "UTF-8", sep = ",")

dataCtr<-na.omit(dataCtr)

dataCtr$pN<- ifelse(dataCtr$pN, "Yes", "No")

dataCtr$age <- as.factor(dataCtr$age)

dataCtr$sex <- as.factor(dataCtr$sex)

dataCtr$type <- as.factor(dataCtr$type)

dataCtr$location <- as.factor(dataCtr$location)

dataCtr$diff <- as.factor(dataCtr$diff)

dataCtr$LVI <- as.factor(dataCtr$LVI)

dataCtr$pT <- as.factor(dataCtr$pT)

dataCtr$length <- as.numeric(dataCtr$length)

dataCtr$pN <- as.factor(dataCtr$pN)

summary(dataCtr)

# Step 2: Create the test dataset

dataCte <- read.table("valid.csv", header = TRUE,,encoding = "UTF-8", sep = ",")

dataCte<-na.omit(dataCte)

dataCte$pN<- ifelse(dataCte$pN, "Yes", "No")

dataCte$age <- as.factor(dataCte$age)

dataCte$sex <- as.factor(dataCte$sex)

dataCte$type <- as.factor(dataCte$type)

dataCte$location <- as.factor(dataCte$location)

dataCte$diff <- as.factor(dataCte$diff)

dataCte$LVI <- as.factor(dataCte$LVI)

dataCte$pT <- as.factor(dataCte$pT)

dataCte$length <- as.numeric(dataCte$length)

dataCte$pN <- as.factor(dataCte$pN)

summary(dataCte)

#

set.seed(123)

logloss=trainControl(method="repeatedcv",

number=10,

repeats=5,

classProbs=TRUE,

savePredictions=TRUE,

summaryFunction = mnLogLoss,

sampling = "rose"

)

bootstrap <- trainControl(method="boot632", number=1000,returnResamp = "all",

classProbs = TRUE, summaryFunction = twoClassSummary, savePredictions = TRUE, sampling = "rose")

#train EL model with hyperparameters according to ‘tgrid’

tuningmodel<- train(pN ~., data=dataCtr, method = "glmnet", trControl = logloss,metric = "logloss",

tuneGrid = expand.grid(alpha = seq(0.02,0.4,by=0.01),lambda = seq(0.001,0.1,by = 0.01)))

tuningmodel$bestTune

###Trains the final model

FinalEL<-train(pN ~., data=dataCtr, method = "glmnet", trControl = bootstrap,metric = "ROC",

tuneGrid = expand.grid(alpha = tuningmodel$bestTune$alpha,lambda = tuningmodel$bestTune$lambda))

###Returns the apparent AUC from the final EL model

getTrainPerf(FinalEL)

###Trains Random Forest Model with hyperparameters according to ‘tgrid’

tgrid<-expand.grid(

.mtry=1:10

)

RFModel<-train(pN ~., data=dataCtr, method="rf", num.trees=1000, na.action=na.pass,replace=TRUE,

trControl= logloss, tuneGrid=tgrid, metric ="logloss")

RFModel$bestTune

###Trains the final Random Forest model

tgrid2<-expand.grid(

.mtry=RFModel$bestTune$mtry

)

FinalRF<-train(pN ~., data=dataCtr, method="rf", num.trees=1000, na.action=na.pass,replace=TRUE,

trControl= bootstrap, tuneGrid=tgrid2, metric ="ROC")

###Returns the apparent AUC from the final RF model

getTrainPerf(FinalRF)

####Trains XGB Model according to tuning parameters in ‘tune_grid’Hosmer-Lemeshow test

cl<-makePSOCKcluster(detectCores(logical=FALSE)-1)

registerDoParallel(cl)

tune_grid <- expand.grid(

nrounds = seq(from = 90, to = 110, by = 5),

eta = c(0.025, 0.05,0.075),

max_depth = c(1.5, 2,2.5),

gamma = c(0,0.05,0.10),

colsample_bytree = c(0.7,0.8,0.9),

min_child_weight = c(1.5,2,2.5),

subsample = c(0.35,0.40,0.45)

)

xgb_tune <-train(pN ~.,

data=dataCtr,

method="xgbTree",

trControl=logloss,

tuneGrid=tune_grid,

verbose=T,

metric="logloss"

)

####Returns Final XGB model

xgb_tune$bestTune

final_grid <- expand.grid(

nrounds = xgb_tune$bestTune$nrounds,

eta = xgb_tune$bestTune$eta,

max_depth = xgb_tune$bestTune$max_depth,

gamma = xgb_tune$bestTune$gamma,

colsample_bytree = xgb_tune$bestTune$colsample_bytree,

min_child_weight = xgb_tune$bestTune$min_child_weight,

subsample = xgb_tune$bestTune$subsample

)

FinalXGB <-train(pN ~.,

data=dataCtr,

method="xgbTree",

trControl=bootstrap,

tuneGrid=final_grid,

verbose=T,

metric="ROC")

###Stops parallel processing

stopCluster(cl)

registerDoSEQ()

###Returns the apparent AUC from the final XGB model

getTrainPerf(FinalXGB)

#Ensembles the Models into one model using linear blend

EnsList<-caretList(pN ~.,data=dataCtr, trControl=logloss,

tuneList=list(

xgbTree=caretModelSpec(method="xgbTree", tuneGrid=final_grid),

rf=caretModelSpec(method="rf", num.trees=1000,tuneGrid=tgrid2),

glmnet=caretModelSpec(method="glmnet",tuneGrid = expand.grid(alpha = FinalEL$bestTune$alpha,lambda = FinalEL$bestTune$lambda)

)))

FinalEns<-caretStack(

EnsList,

method="glm",

metric="ROC",

trControl=bootstrap

)

print(FinalEns)

print(FinalRF)

print(FinalEL)

print(FinalXGB)

###Returns the apparent ROC and Calibration Chart

getTrainPerf(FinalEns)

#train set ROC

Xr<-predict(FinalEL,newdata = dataCtr,type="prob")

class(Xr[2,2])

X1r<-Xr[,2]

X2r<-as.numeric(dataCtr[,9])-1

X3r<-cbind2(X1r,X2r)

XRr<-predict(FinalRF,newdata = dataCtr,type="prob")

X1Rr<-XRr[,2]

X2Rr<-as.numeric(dataCtr[,9])-1

X3Rr<-cbind2(X1Rr,X2Rr)

X3Rr

XXr<-predict(FinalXGB, newdata = dataCtr,type="prob")

XXr

X1Xr<-XXr[,2]

X2Xr<-as.numeric(dataCtr[,9])-1

X3Xr<-cbind2(X1Xr,X2Xr)

XEr<-predict(FinalEns,newdata = dataCtr,type="prob")

XEr

X1Er<-1-XEr

X2Er<-as.numeric(dataCtr[,9])-1

X3Er<-cbind2(X1Er,X2Er)

par(mfrow=c(1,1))

FinalModelROC <- plot.roc(X3r[,2], X3r[,1],

main="Elastic Net",

grid=TRUE,auc=TRUE,print.auc=TRUE, percent=TRUE,

xlim=c(100, 0), ylim=c(0, 100),

xlab="Specificity (%)", ylab="Sensitivity (%)",

ci=TRUE)

ciroccurve <- ci.se(FinalModelROC,specificities = seq(0, 100, 5), boot.n=1000)

plot(ciroccurve, type = "shape", col = "lightgrey")

RFModelROC <- plot.roc(X3Rr[,2], X3Rr[,1],

main="RandomForest",

grid=TRUE, auc=TRUE,print.auc=FALSE, percent=TRUE,

xlim=c(100, 0), ylim=c(0, 100),

xlab="Specificity (%)", ylab="Sensitivity (%)",

ci=TRUE)

ciroccurveR <- ci.se(RFModelROC,specificities = seq(0, 100, 5), boot.n=1000)

plot(ciroccurveR, type = "shape", col = "lightgrey")

XGBModelROC <- plot.roc(X3Xr[,2], X3Xr[,1],

main="XGBoost",

grid=TRUE, auc=TRUE,print.auc=FALSE, percent=TRUE,

xlim=c(100, 0), ylim=c(0, 100),

xlab="Specificity (%)", ylab="Sensitivity (%)",

ci=TRUE)

ciroccurveX <- ci.se(XGBModelROC,specificities = seq(0, 100, 5), boot.n=1000)

plot(ciroccurveX, type = "shape", col = "lightgrey")

EnsModelROC <- plot.roc(X3Er[,2], X3Er[,1],

main="Ensemble",

grid=TRUE, auc=TRUE,print.auc=FALSE, percent=TRUE,

xlim=c(100, 0), ylim=c(0, 100),

xlab="Specificity (%)", ylab="Sensitivity (%)",

ci=TRUE)

ciroccurveE <- ci.se(EnsModelROC,specificities = seq(0, 100, 5), boot.n=1000)

plot(ciroccurveE, type = "shape", col = "lightgrey")

#calibrate.plot

{par(mfrow=c(2,2))

calibrate.plot(X3r[,2],X3r[,1], ylab="Observed Probability", xlab="Predicted Probability",main="Elastic Net")

calibrate.plot(X3Rr[,2],X3Rr[,1], ylab="Observed Probability", xlab="Predicted Probability", main="Random Forest")

calibrate.plot(X3Xr[,2],X3Xr[,1], ylab="Observed Probability", xlab="Predicted Probability", main="XG Boost")

calibrate.plot(X3Er[,2],X3Er[,1], ylab="Observed Probability", xlab="Predicted Probability", main="Ensemble")

}

###Returns the Hosmer-Lemeshow test for the final model with 10 bins

HLFull<-hoslem.test(X3r[,2],X3r[,1],g=10)

HLFull

HLRFull<-hoslem.test(X3Rr[,2],X3Rr[,1],g=10)

HLRFull

HLXFull<-hoslem.test(X3Xr[,2],X3Xr[,1],g=10)

HLXFull

HLEFull<-hoslem.test(X3Er[,2],X3Er[,1],g=10)

HLEFull
